# Trauma Induced Calcium Pyrophosphate Deposition Disease of the Lumbar Spine

**DOI:** 10.1155/2020/3218350

**Published:** 2020-02-10

**Authors:** Indrit Greca, Jihad Ben Gabr, Andras Perl, Stephanie Bryant, Dan Zaccarini

**Affiliations:** ^1^SUNY Upstate University Hospital, Syracuse, NY-13210, USA; ^2^Departments of Medicine, Microbiology and Immunology, Biochemistry and Molecular Biology, State University of New York, UMU, College of Medicine, New York, USA

## Abstract

Inflammatory arthritis, such as pseudogout or otherwise referred to as calcium pyrophosphate (CPP) crystal arthritis or calcium pyrophosphate deposition (CPPD) disease, is characterized by the deposition of crystal formation and deposition in large joints. CPPD is known to affect the elderly population and commonly manifests as inflammation of knees, hips, and shoulders. CPPD disease involving the spine has been infrequently encountered in practice and rarely described in the literature. Here, we describe a case of an 80-year-old female with no known history of inflammatory arthritis who presented with left lower extremity weakness and fall, initially thought to have discitis, later confirming CPPD of the spine through biopsy and ultimately resolution of symptoms with anti-inflammatory agents. Although consisting of different clinical presentations, two other case reports have described CPPD of the spine with similar radiographic findings, to this author's knowledge. With the radiologic similarities, this unique case serves to raise awareness in the medical community and possibly place pseudogout of the spine on the differential list when such cases are encountered. As a result, patients can be initiated on benign anti-inflammatory agents, avoiding invasive testing and unnecessary antibiotic exposure.

## 1. Introduction

Back pain is one of the most common symptoms experienced by an adult which oftentimes leads to medical evaluation. Approximately 84% of adults have experienced some degree of back pain in their lifetime [1]. Etiologies of back pain vary, but the majority of patients with these complaints will not have a definite known etiology [2]. Most common known etiologies appear to be muscular, whereas less than 1 percent of patients with back pain have nonbenign etiologies such as infection or malignancy [3]. Spinal inflammatory arthritis, such as acute CPP crystal arthritis, is not a typical etiology that would present as a differential of back pain and is not often thought of as a possible diagnosis. Acute CPP crystal arthritis is the deposition of calcium pyrophosphate dihydrate crystals in connective tissues, giving rise to inflammatory arthritis [4]. Etiology of acute CPP crystal arthritis is idiopathic, but joint trauma has been a linked factor [5]. The deposition of crystals most commonly affects the knees and the wrists. Here, we present an 80-year-old female with no past history of pseudogout who presented with back pain presumed to be of infectious origin but was ultimately found to have pseudogout of the lumbar spine. The purpose of this documentation is to bring awareness to pseudogout affecting the spine, which would otherwise be misdiagnosed as infection in patients with undistinguishable radiographic evidence, and expose these patients to unnecessary antibiotic treatment.

## 2. Case Presentation

80-year-old female with known medical history of hypothyroidism, gastroesophageal reflux disease, and hypertension presented to our hospital with left leg weakness with tingling prompting a fall, associated with progressive back pain. The patient started having back pain about one year prior to presentation after suffering a mechanical fall down a flight of stairs. At the time, she did not seek medical attention and pain improved with conservative treatment. However, six months ago the patient started having intermittent low back pain radiating to left buttock, for which she saw her primary care physician (PCP). Her PCP prescribed a short course of tramadol of unknown dosage and frequency. Approximately one month prior to presentation, the patient started experiencing exacerbations of her back pain associated with radiculopathy on bilateral lower extremities. She self-treated with aspirin 325 milligrams (mg) every 8 hours as needed along with Tylenol 650 mg every 6 hours as needed. On the day of hospitalization, the patient experienced severe left lower extremity numbness subsequently leading to her fall. She otherwise denied urinary or bowel incontinence, perineal paresthesia, fevers, chills, dizziness, palpitations, loss of consciousness, rashes, unintentional weight loss, and headaches.

On day 1 of examination, the patient was in no acute distress but appeared uncomfortable. The temperature was 36.4°C, the blood pressure was 104/65 mmHg, the pulse was 54 beats per minute, the respiratory rate was 16 breaths per minute, the oxygen saturation was 98% while she was breathing ambient air, and body mass index was 32.07 kilograms per meter squared (kg/m^2^). Physical examination revealed pupils were equal, round, and reactive to light. Heart sounds were normal, with regular rate and rhythm. Lung sounds were clear bilaterally. The abdomen was soft and nontender. Musculoskeletal examination was remarkable for mild point tenderness of the lumbar spine. Rectal tone was intact. Strength and sensation were intact on bilateral upper and lower extremities. Patellar reflexes were plus one bilaterally. No clonus was noted, and Babinski sign was negative. There were no abrasions or discoloration of the skin noted on the paraspinal area. The paraspinal area was clean and nonerythematous. The patient was found to have abdominal fold and bilateral groin wounds that appeared moist and pale pink with some partial thickness and skin breakdown. There appeared to be some chronic skin changes with dark skin discoloration to the periphery as well.

**Figure 1:**
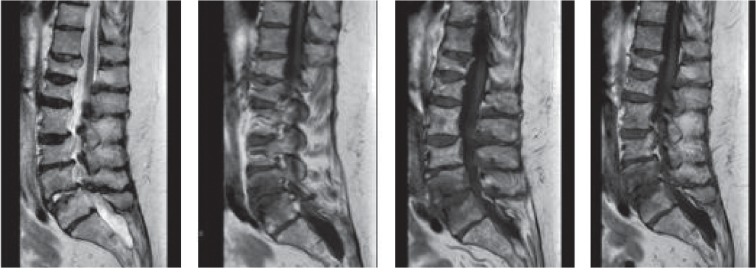
Axial and sagittal T1 and T2-weighted and coronal and sagittal short-TI inversion recovery (STIR) images of the lumbar spine without intravenous contrast administration. Axial and sagittal T1-weighted images were then acquired following intravenous administration of 80 mL of Gadavist. The image shows vertebral body edema and enhancement most evident at the L4L5 level. Paravertebral soft tissue thickening is seen at the L5-S1 level, with extension into the left neural foramina. Findings are suggestive of osteomyelitis/discitis.

Laboratory studies showed blood urea nitrogen of 32 mg/dL (reference range: 8–23 mg/dL; reference range is provided in the parentheses in the following laboratory studies), creatinine of 1.23 mg/dL (0.4–1.0 mg/dL), corrected calcium of 11.1 mg/dL (8.8–10.2 mg/dL), and a white blood cell count of 11.8 K/*μ*L (4–10 K/*μ*L). The rest of the basic laboratory studies were within normal range. The initial image of choice ordered was a computerized tomography (CT) scan, which showed advanced degenerative joint disease with severe spinal stenosis at the level of the lumbar 4-lumbar 5 (L4L5) spinal segment. Orthopedic spine specialty service was consulted and recommended to administer intravenous (IV) Decadron 6 milligrams every 6 hours and obtain a magnetic resonance imaging (MRI) for further evaluation of the abnormality noted on CT scan. MRI of the spine was completed on day 2, which depicted vertebral body edema and enhancement most evident at the L4L5 level and paravertebral soft tissue thickening seen at the L5-S1 level concerning of osteomyelitis/diskitis. (Figure 1). MRI also confirmed severe central canal stenosis at the L4L5 level with crowding of the cauda equina secondary to disk bulging and osteoarthropathy. Due to concerns of possible osteomyelitis of the spine, the patient was started empirically on vancomycin 2 grams every 12 hours and piperacillin/tazobactam 3.375 grams every 6 hours. A neurosurgery consult was requested.

Neurosurgery evaluated the patient and were concerned about the severe central canal and neuroforaminal stenosis as depicted by the imaging. They stated that the patient could have been a candidate for a L45 transforaminal lumbar interbody fusion (TLIF) as it appeared that there were significant sclerotic changes at this spinal level causing neurological deficits. However, further investigation needed to be done to determine the underlying etiology. Decadron was continued in the interim, and the patient's symptoms continued to improve. Due to stabilizing neurological physical examination, neurosurgery recommended biopsy of site to officially rule out osteomyelitis/discitis. In this setting, inflammatory markers were obtained. Erythrocyte sedimentation rate (ESR) was found to be 13 mm/hr (<30 mm/hr), and C-reactive protein was found to be 2.9 mg/L (<8.0 mg/dL). With normal inflammatory markers, antibiotics were discontinued on day 2 as likelihood of osteomyelitis was low.

**Figure 2:**
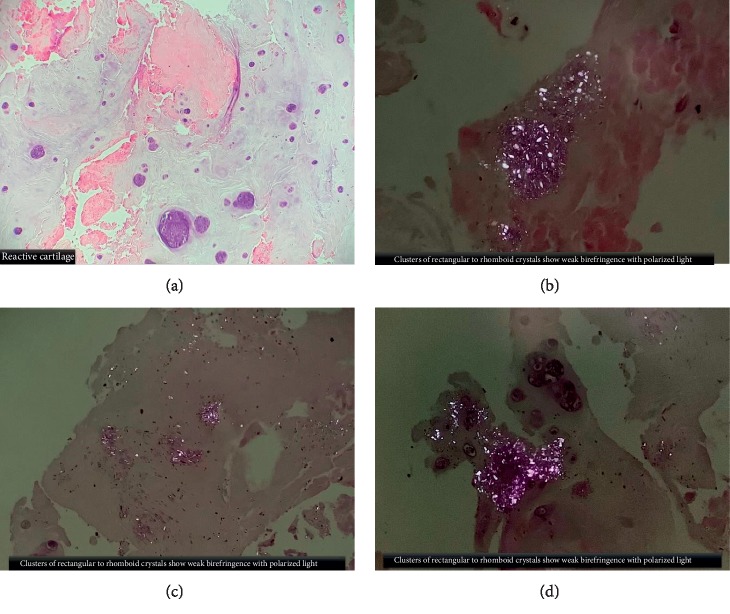


On day 3, interventional radiology (IR) image-guided biopsy and aspiration of the L4L5 vertebral bone was done. Final pathology report showed fragments of cartilage with calcium pyrophosphate crystal deposition supporting a diagnosis of acute CPP crystal arthritis of the lumbar spine (Figures 2(a)–2(d)). Biopsy cultures for infection were negative. Blood cultures resulted as no growth. Given these findings, rheumatology service was consulted. The patient was started on colchicine 0.6 mg daily. Steroids were stopped on day 7 as initial symptoms improved. Rheumatology service also requested X-ray imaging of bilateral hands, wrists, and knees to assess for CPPD involvement. Imaging supported degenerative joint disease but did now show any signs of CPPD disease at these joints. The patient was eventually transferred to a rehabilitation center for the deconditioning. She was instructed to follow up with rheumatology services on an outpatient basis; however, the patient missed her appointment.

## 3. Discussion

Spinal pseudogout is a rare entity, with few case reports described in the literature, most of which depict pseudogout of the cervical region [6]. Based on literature review, the most commonly affected section of the spine has been reported to be the cervical spine [6, 7]. A study was conducted in the Rheumatology Department of Lariboisiere Hospital in Paris which attempted to find the prevalence of spinal involvement in patients with established diagnoses of widespread CPPD. It was noted that 24% of patients who were hospitalized with CPPD had spinal involvement, mostly affecting the cervical and lumbar spine; however, they had widespread CPPD disease [6]. In this case report, the patient had no previous history of pseudogout and had no other joint involvement after thorough investigation.

The patient in this encounter obtained trauma at the site of crystal deposition prior to this hospitalization, which was rendered as the predisposition factor contributing to the formation of pseudogout. To the author's knowledge, there are no known case reports published which depict pseudogout formation of the spine after obtaining direct trauma. One case report by Bridges et al. described a 66-year-old female who underwent L2–5 laminectomies, bilateral foraminotomies, and L4–5 transforaminal lumbar interbody fusion with L4–5 posterior instrumented fusion for spinal stenosis and subsequently was found to have CPPD of the cervical and thoracic spine [8]. However, the patient had surgical intervention of the lumbar region, not involving the particular affected sites.

MRI is typically the method of choice when evaluating for pathologies of soft tissue, bone, and joints. MRI features of osteomyelitis or discitis commonly include descriptions of edema and disc degeneration of the affected site, according to a retrospective review of the database of spinal infections [9, 10]. An article written by Mechri et al. describes imaging features of malignant tumors of the spine [11]. According to this article, spinal tumors will be demonstrated typically as lytic lesions, well-formed tumors, or low to high signal intensity on T2-WI [11]. These findings are characteristic and are not to be confused with typical findings of pseudogout of the spine. Although there are limited data on the presentation of spinal pseudogout, a common theme of misdiagnosis of discitis or osteomyelitis has been reported due to common presenting symptoms of infection and image findings of edema, enhancement, and tissue thickening. Two case reports have been published which describe elderly patients presenting with fever, back pain, and subsequent elevated inflammatory markers [8, 12]. Moreover, the cases also describe similar findings on imaging, which further support including spinal pseudogout on the differential when such findings are recovered. In this case report, the MRI findings depicted vertebral body edema and enhancement, leading clinicians to the initial misdiagnosis of vertebral body osteomyelitis/discitis. The aim of this document along with the abovementioned articles is to establish radiological classification of pseudogout of the spine.

The case report by Bridges et al. depicts a case in which the clinical presentation and image findings were initially thought to be of infectious origin, as was the case in our patient. In this case report, the patient had surgical intervention of the spine and subsequently presented to the ED with complaints of fever, chills, and back spasms. The patient was found to have elevated inflammatory markers and had an MRI subsequently, which was concerning for infection.

**Figure 3:**
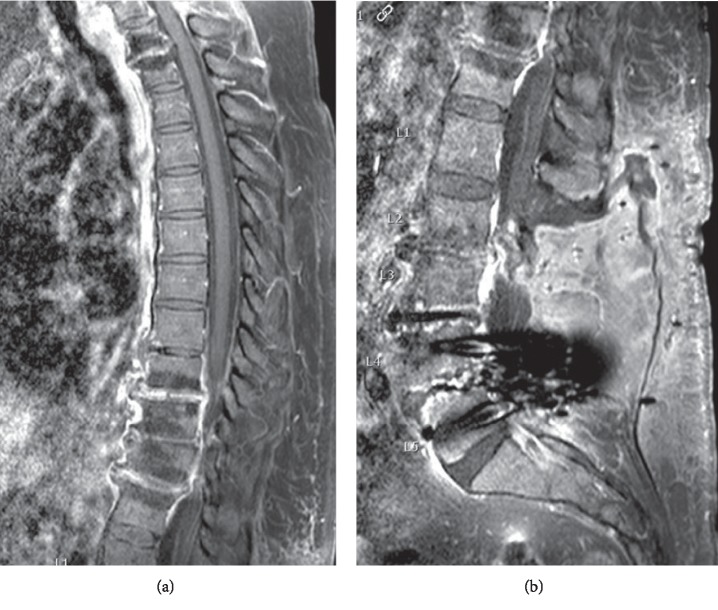
Thoracic (a) and lumbar (b) spine MR images demonstrate some enhancement within the C7–T1 acet joints as well as the T1–2 and T9–12 intervertebral discs. There is also ventral epidural enhancement from T9 to T12 and some enhancement within the lumbar surgical site.

Antibiotics were started empirically regardless of sterile biopsy results. As the patient's condition progressed on antibiotics, a full-spine MRI was done, which demonstrated “marked increase in the enhancement of the C (cervical) 7–T1 facet joints with extension into the paraspinal soft tissues and the posterior epidural space, resulting in spinal cord compression” (Figure 3). The patient underwent biopsy once again, which remained sterile; however, on further microscopic review, there was evidence of pseudogout. This patient shortly after developed widespread CPPD which further supported the diagnosis of spinal pseudogout. The patient's symptoms resolved after initiation of the interleukin-1 inhibitor [8].

**Figure 4:**
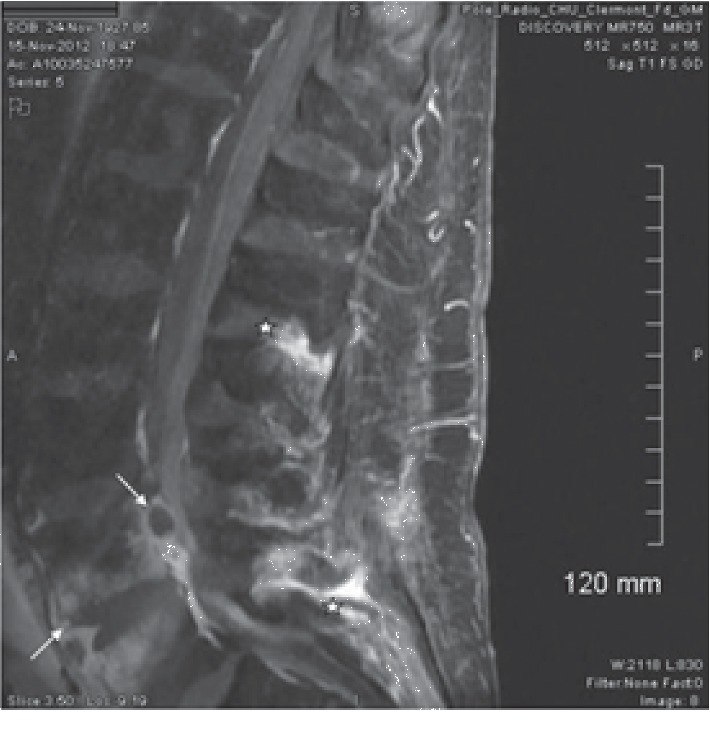
MRI of the lumbar spine (T1 fat sat + gadolinium): the gadolinium-enhanced sagittal T1-weighed MR image shows peripheral enhancement of L4-L5 disc and marrow edema extending from the L4 and L5 vertebrae with anterior epidural abscess-like spondylodiscitits (arrow). The MR image shows inflammation with enhancement of L1-L2 and L4-L5 zygapophysial joints (stars).

Another case report documented by Grobost et al. illustrated a case of pseudogout of the lumbar spine presenting with clinical and image findings concerning for spondylodiscitis and epidural abscess with resultant lumbar percutaneous needle biopsy supporting pseudogout instead (Figure 4) [12]. The authors describe a case of an elderly male patient with recurrent back pain and history of CPPD. The patient was readmitted for back pain and obtained imaging prior to biopsy. MRI findings in this case depicted “peripheral enhancement of L4L5 disc and marrow edema extending from L4 and L5 vertebrae with anterior epidural abscess-like spondylodiscitis,” as well as “inflammation with enhancement of L1L2 and L4L5 zygapophysial joints.” The reading of this MRI shows similarities from the previous mentioned case by Bridges et al. as well as our case.

Both case reports demonstrated similar findings on imaging, depicting enhancement of the spinal region with extension into the paraspinal soft tissues. The MRI findings in our patient also showed enhancement at the affected spinal level with paravertebral soft tissue thickening. These depicted similarities, along with imaging analysis, can serve to establish guidelines in differentiating back pain. The manifestation of inflammatory joints in pseudogout originated from the deposition of calcium pyrophosphate dihydrate crystals in soft tissue. The approach to take in such scenarios, as depicted in the above cases, would be to try first-in-line treatments with anti-inflammatory agents, such as NSAIDs, Colchicine, and/or steroids. Although rare, it is crucial to suspect spinal pseudogout as its clinical manifestations can be debilitating, often leading to spinal cord compression, acute nerve compression, and spinal stenosis, amongst many other syndromes [13, 14]. Suspecting pseudogout in elderly patients with similar clinical presentation, and image findings can enable the medical community to initiate treatment promptly and avoid predisposing patients to unnecessary invasive procedures and exposure to antibiotics.
